# British Provincial Medical and Surgical Association

**Published:** 1849-01

**Authors:** 


*British Provincial Medical and Surgical Association.*—Professor Wood, of Philadelphia, attended the meeting of this Association at Bath, in August last, in behalf of the American Medical Association, and on being toasted, delivered the following address:

"Mr. Chairman and gentlemen: It is with emotions, that, I fear, may prevent the expression of all I feel on this occasion, that I rise to thank the present company for the honor they have done me, and more especially for the honor they have done my country through me. (*Cheers.*) Science, sir, is universal—it is born of the necessities of our nature, and its objects are to promote the good of mankind; and therefore, its cultivators in every clime ought to be brethren. (*Hear, hear.*) If it be true of the great system of science, that by strong attraction, all parts are kept together, it is still more so in its inferior or subordinate relations. Like the planets of the solar system, though constituting a great mass of the stupendous whole, there is a closer affinity to each other than to other parts; so we, as scientific men, have a connexion, but as medical men, we are bound together by a stronger link. We, of America, have the same origin with you, which binds us by a closer tie. We have the same climate, the same literature, for we draw all our knowledge, (our scientific knowledge I mean,) from almost the same sources; and therefore, how important it is that we should cultivate the spirit of brotherhood. (*Hear, hear.*) Standing here as the representative of the intelligence of America, in relation to this country, for I believe I may add, that in our land, amoug the well informed, there is a universal prevalence of this feeling towards the mother country, I say, sir, that we are extremely anxious to cultivate this reciprocal spirit from those in the highest ranks of knowledge to the lowest grades of society. (*Cheers.*) We have, gentlemen, the fairest race on earth. We have also the impulse of the Welsh or Celts, the steadiness of the Saxon or German character, and the energy of the Norman race; and we shall go on, unless interrupted by ambition, until all parts of the earth are impregnated with the Saxon religion, and to such an end I am bound to wish a speedy consummation. (*Cheers.*)—*New York Jour. of Med.*

